# Belief in a Just World and Moral Personality as Mediating Roles Between Parenting Emotional Warmth and Internet Altruistic Behavior

**DOI:** 10.3389/fpsyg.2021.670373

**Published:** 2021-10-06

**Authors:** Ye Zhang, Liang Chen, Yumeng Xia

**Affiliations:** ^1^College of Educational Science, Shenyang Normal University, Shenyang, China; ^2^School of Marxism, University of Science and Technology Liaoning, Anshan, China; ^3^The Communist Youth League, State Grid Anshan Electric Power Supply Company, Anshan, China

**Keywords:** parental emotional warmth, internet altruistic behavior, mediation effect, belief in a just world, moral personality

## Abstract

This study explores the influence of parental emotional warmth (PEW) on college students’ Internet altruistic behavior (IAB), and the mediating roles of personal belief in a just world (PBJW) and positive moral personality traits (PMPT). A total of 893 college students were assessed using questionnaires. Results: (1) PEW, PBJW, PMPT, and IAB are positively correlated with each other; (2) PEW can directly predict the IAB of college students; and (3) PEW can indirectly predict IAB through the mediating effect of PMPT and PBJW-PMPT. PBJW and PMPT account for 22.79% of the total influence of PEW on IAB.

## Introduction

Internet altruistic behavior (IAB) refers to some voluntary behaviors displayed on the Internet for the benefit others and society, without expectation of anything in return. These include reminding, supporting, sharing information with, and guiding others online (Zheng et al., 2011). As cyberspace provides anonymity to users, people who seek help on the Internet rarely disclose personal information, which they otherwise would offline. They are also more likely to seek help from individuals online than from those around them (Sproull et al., 2005). As the bystander effect does not exist online, IAB occurs more frequently than altruistic behavior in real life (Wang et al., 2021). Those who provide the help as well as the recipients of this help are considerably happier because of altruistic behavior (Takebe and Murata, 2017). Therefore, this study investigates the mechanism of IAB, specifically in college students.

Social learning theory assumes that children’s extensive adaptive outcomes are influenced by early parenting experiences and can be continually enhanced during growth (Liu et al., 2003). In particular, families with positive parenting styles provide their children with an array of encouragement, support, and help, which could be helpful for the children’s development. In this study, we explore the relationship between parental emotional warmth (PEW) and individual’s IAB through two main development areas: cognitive [personal belief in a just world (PBJW)] and personality [positive moral personality traits (PMPT)]. Considering previous studies, PBJW and PMPT are potential mediating mechanisms that associate PEW with individual behavioral development (Nudelman, 2013; Wang et al., 2021). Therefore, while considering IAB as an adaptive consequence in college students, we investigate the association between PEW and IAB, and the mediating roles of PBJW and PMPT in this association.

### PEW and IAB

Parenting style is the behavioral tendency that parents show while educating and raising children. It is comprehensively displayed in parents’ educational concepts and behavior (Jiang, 2004). Social learning theory indicates that altruistic behavior is learned and that this influence lasts until adulthood (Liu et al., 2003). Thus, parenting style is an important factor that cannot be disregarded in individual altruistic behavior (Li, 2000). PEW, however, is a positive parenting style in which, parenting behaviors are supportive, reactive, and consistent and include timely, sensitive responses to children’s needs (Gauvain and Huard, 1999). PEW can promote a strong sense of self-worth and security, psychological well-being, helping behavior, and other positive outcomes (Wolfradt and Engelmann, 2003). Therefore, when using the Internet, college students who grow up under active parenting styles are likely to be active in solving problems that they encounter online, such as helping and reminding others. Hence, this study assumes that PEW is positively correlated with IAB.

### Belief in a Just World as a Mediating Variable

According to just-world theory, belief in a just world (BJW) is when “people believe that the world they live in is a just world, where they will be treated fairly. Good people will be rewarded, bad people will be punished, and everyone will get what they deserve” (Lerner and Miller, 1978; Lerner and Simmons, 1966). Dalbert (1999) divided BJW into two dimensions: PBJW and general belief in just world (GBJW). GBJW operates on the belief that the world is generally fair, and people will get what they deserve. GBJW is commonly associated with less helping behavior and indifferent, delinquent attitudes toward social suffering (Bègue and Muller, 2006; Sutton and Winnard, 2007; Khera et al., 2014). In contrast, PBJW is mainly concerned with self-related justice, such as the belief that one is being treated fairly, and is linked with more prosocial behavior (Hafer and Sutton, 2016; Wang et al., 2021). Thus, individuals with high levels of PBJW sympathize with those seeking help and are likely to aid them.

The socialization theory developmental model suggests that the quality of social interpersonal relationships among children and adolescents has a significant impact on the internalization of their beliefs and values (Grusec and Goodnow, 1994; Dalbert, 1999). A positive parenting style is conducive to strengthening cooperation and sharing between individuals, enhancing mutual trust, and making individuals feel that they are being treated fairly. Previous studies have generally explored the influence of parenting style on adolescents’ beliefs in a just world. For example, Dalbert and Sallay (2004) showed that individuals living in an emotionally oriented family atmosphere usually have a stronger belief in a just world. Therefore, the current study assumes that PBJW plays a mediating role in the relationship between PEW and IAB.

### PMPT as a Mediating Variable

Moral personality is the moral dimension of personality and the overall organization of moral cognition, emotion, and behaviors formed by individuals during the socialization process (Wang and Guo, 2013). It is the unity of an individual’s inner qualities and external moral behaviors. The moral personality of college students includes both positive and negative moral personality traits. PMPT mainly include benevolence, faith, respect, integrity, selflessness, honesty, and diligence-frugality, whereas negative moral personality traits mainly include unrighteousness, utilitarian, indulgence, deceit, and aggression (Wang and Guo, 2013). Parenting styles play an important role in the internalization of such moral values by children and adolescents (Fatima et al., 2020). A meta-analytic review research has also shown that PEW is significantly positively correlated with children’s positive personality dispositions (Khaleque, 2013). Furthermore, cognitive factors can influence the formation and development of personality (Wang, 2009). Studies have shown that PBJW is also significantly positively correlated with personality traits, such as extroversion and agreeableness (Nudelman, 2013).

Social exchange theory indicates that helpers actually receive an internal reward, although external benefits are lacking (Homans, 1958). Helpers affirm self-worth through altruistic behavior and experience happiness from self-realization. Studies have found that individual moral personality traits are closely related to altruistic behavior (Li, 2000). PMPT is significantly and positively correlated with moral behavior. The higher the positive moral personality level of an individual, the more likely they will be to engage in altruistic behavior (Wang, 2009). Zheng and Gu (2012) found that personality traits such as extroversion, conscientiousness, and openness are significantly positively correlated with IAB. Therefore, this study presents the following assumptions: (1) PMPT is positively correlated with IAB, (2) PMPT plays a mediating role between PEW and IAB, and (3) PBJW and PMPT play a chain mediating role between PEW and IAB.

**Figure 1:**
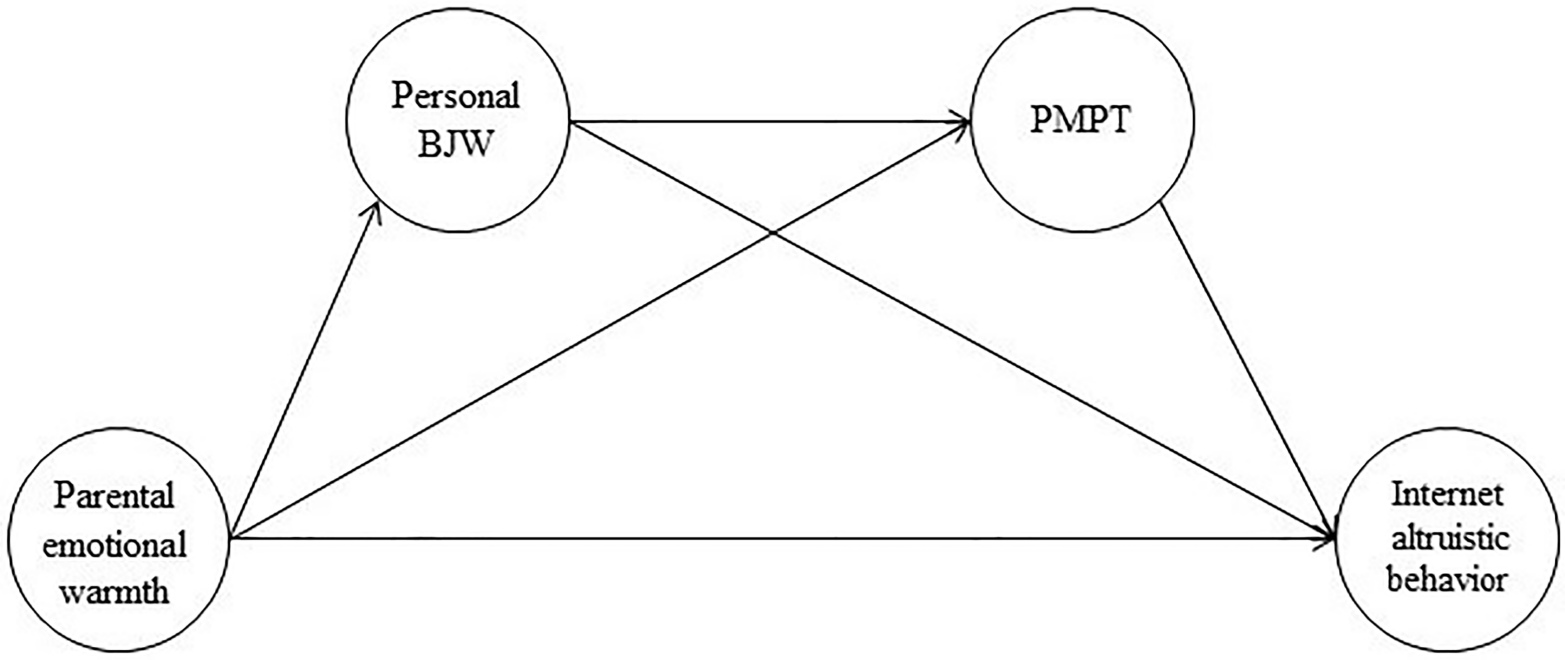
The proposed mediation model.

In summary, the present study establishes a hypothetical model to explore the influence of PEW on college students’ IAB, and the mediating roles of PBJW and PMPT (see Figure 1).

## Materials and Methods

### Participants

The study protocol was approved by the Research Ethics Committee of the authors’ affiliation. The stratified cluster random sampling method was used to select 893 students from freshman to senior years from two universities in Liaoning as participants. A total of 1,000 questionnaires were distributed. After the collection, 893 valid questionnaires were obtained, with an effective recovery rate of 89.3%. Participants included 528 men (59.1%) and 365 women (40.9%). Their ages ranged from 17 to 23years, with an average age of 20.44 (*SD*=1.44).

### Measures

#### Swedish Acronym for Short Version of Egna Minnen Beträffande Uppfostran

Perris et al. (1980) developed a new inventory Egna Minnen Beträffande Uppfostran (EMBU) to assess memories of parental rearing behavior. The simplified Chinese version of Swedish acronym for short version of Egna Minnen Beträffande Uppfostran (s-EMBU) was revised by Jiang et al. (2010), including three dimensions: parental emotional warmth (eight items), parental rejection (seven items), and parental overprotection (six items). The parental emotional warmth subscale was used in this study. A four-point scoring scale was used, where 1 represents “never,” and 4 represents “always.” The average sum score of the two parents in each dimension was calculated. The Cronbach’s α coefficients of the mother and father emotional warmth subscales are 0.790 and 0.795, respectively.

#### Belief in a Just World Scale

Dalbert (1999) developed the BJW scale. The Chinese version of the BJW scale, revised by Wu et al. (2011), was adopted in this study. The questionnaire consists of 13 items. Participants responded on a six-point scale, in which 1 indicates “completely disagree,” and 6 indicates “completely agree.” The questionnaire comprises two subscales: GBJW (six items) and PBJW (seven items). The PBJW subscale was used in this study. The Cronbach’s α coefficient of the PBJW subscale was 0.874.

#### Undergraduates’ Moral Personality Adjective Evaluation Questionnaire

Based on the lexical hypothesis of personality research, Wang (2009) used moral personality trait analysis to examine the structure of college students’ moral personality. First, adjectives describing the moral personality characteristics were determined. Second, the glossaries were simplified, and unbiased samples were selected. Third, the college students rated the degree to which each adjective described themselves or others, and then, the structure was determined through factor analysis. Finally, the undergraduates’ moral personality adjective evaluation questionnaire (UMPAEQ) was compiled by Wang (2009).

The UMPAEQ includes 72 terms that mainly pertain to seven dimensions of undergraduates’ moral personality: benevolence (nine items), faith (seven items), respect (three items), integrity (four items), selflessness (four items), honesty (six items), and diligence-frugality (two items), which are PMPT. Immorality (36 items), a negative moral personality trait. The PMPT subscales were also used in this study. Participants rated each item on a five-point Likert scale (1=strongly disagree; 5=strongly agree). The Cronbach’s α reliability coefficients of six subscales were acceptable (0.683–0.795), excluding the respect subscale (0.581).

#### The Internet Altruistic Behavior Scale

The IAB scale compiled by Zheng et al. (2011) was adopted for this study. The IAB questionnaire contains 26 items. A five-point Likert scale was used (1=None, 5=Always), with a higher score indicating greater IAB. The questionnaire included four subscales: network support, network guidance, network sharing, and network reminders. The Cronbach’s α coefficient of the total scale was 0.937. The Cronbach’s α coefficient of each subscale was 0.872, 0.824, 0.783, and 0.795, respectively.

### Data Analysis

SPSS 23.0 was used for the descriptive statistics, reliability, and correlation analyses. The series mean was used to deal with missing values. Mplus 8.1 was used to perform a mediation analysis of the structural equation modeling (SEM). Considering the large number of items in the s-EMBU, UMPAEQ, and IAB scales, this study parceled the items of each scale according to the item parceling strategies to simplify the structure of the model (Ran et al., 2007).

## Results

### Common Method Bias Test

To avoid common method biases in the self-assessment analysis and increase the authenticity of the subjects’ responses, all questionnaires were filled anonymously during the sampling test. The exploratory factor analysis of 91 items of the four scales was carried out using Harman’s single-factor test. A total of 19 factors with characteristic roots greater than 1 were extracted. The explanatory power of the first factor was only 15.897%, which is less than 40% of the judging criteria. Therefore, a common method bias was not observed in this study.

### Correlation Analysis Among the Variables

Descriptive statistics and correlation analyses were performed. The correlation analysis results showed significant positive correlations among PEW, PBJW, PMPT, and IAB (see Table 1).

**Table 1:** Correlation matrices in key variables (*N*=893).

Variables	*M*	*SD*	1	2	3	4
1 Parental emotional warmth	2.736	0.506	1			
2 Personal belief in a just world	4.172	0.835	0.317^**^	1		
3 Positive moral personality traits	4.172	0.835	0.329^**^	0.259^**^	1	
4 Internet altruistic behavior	3.758	0.518	0.190^**^	0.128^**^	0.198^**^	1

** *p<0.01*.

### Analysis of Mediating Effects

This study used a bootstrapping and Markov chain Monte Carlo (MCMC) method to test the mediating effects of PBJW and PMPT. First, the significance of the total effect was examined. In this study, the total effect of PEW on IAB was 0.215 (c), with a significant total effect coefficient (*p*<0.001), and almost fitting indices of the total effect model are accepted (*χ*^2^=67.932^***^, *df*=8, CFI=0.979, TLI=0.960, SRMR=0.024, and RMSEA=0.092).

**Figure 2:**
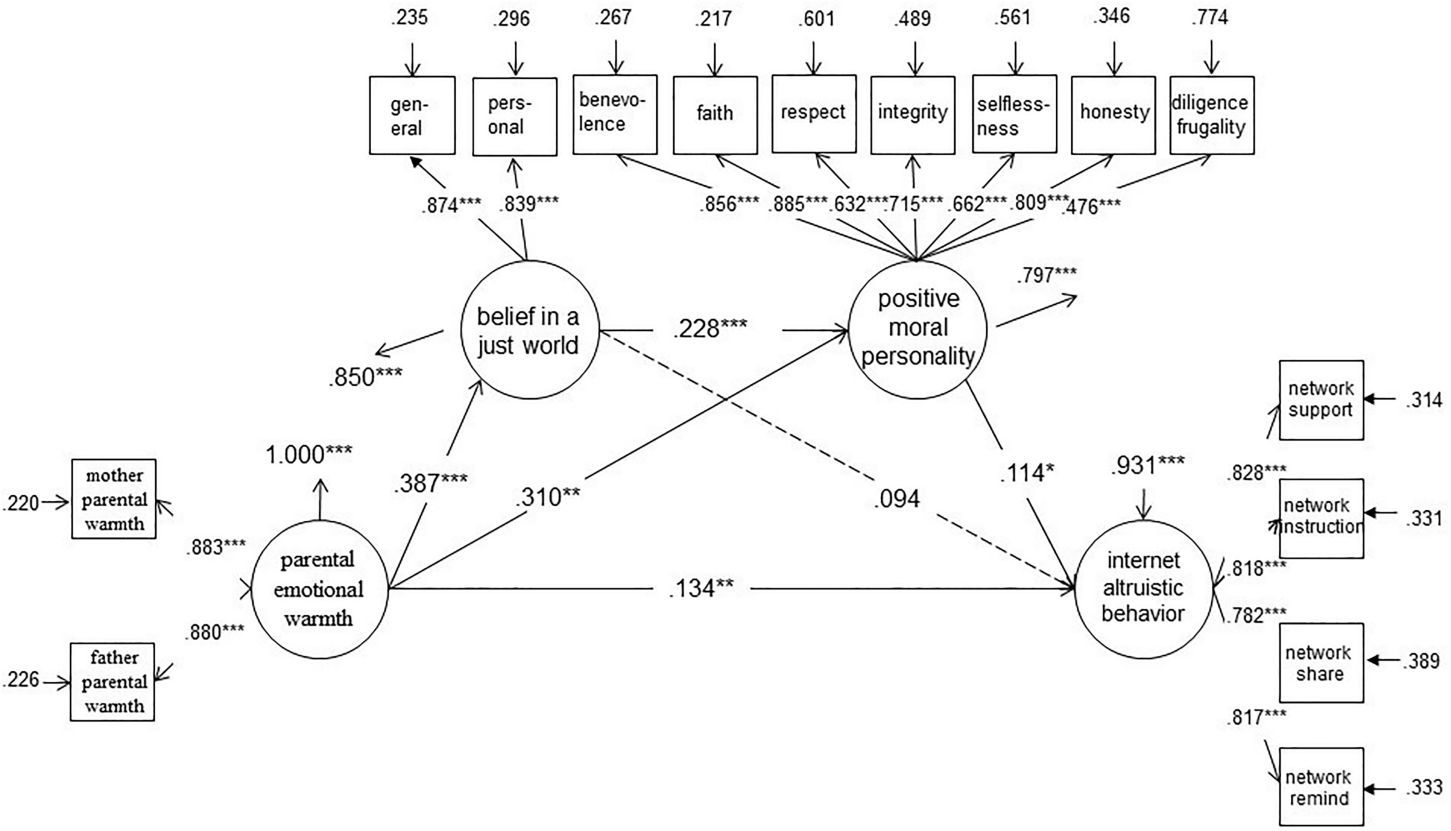
A mediation model of the personal belief in a just world (PBJW) and positive moral personality traits (PMPT) between parental emotional warmth and Internet altruistic behavior (IAB; *N*=893). *p<0.05, **p<0.01, ***p<0.001

Second, the significance of each path coefficient of the mediation model was checked. In this study, the mediating model A was constructed (see Figure 2). The fitting indices of the model were good (*χ*^2^=695.565^***^, *df*=164, CFI=0.943, TLI=0.934, SRMR=0.040, and RMSEA=0.060). SEM showed that the path coefficient of PBJW → IAB was 0.053 (*p*>0.05). The other path coefficients and the normalized factor loads of the observed variables reached the significance level (see Figure 2), thereby indicating that the model was standard. Therefore, PEW has a direct predictive effect on the IAB, while PMPT and PBJW-PMPT play mediating roles in the relationship between PEW and IAB.

Lastly, the CI of path coefficients was estimated, and a total of 1,000 samples were selected through random sampling (see Table 2). In model A, the mediating effect of PBJW and PMPT on PEW and IAB was 0.049, which accounted for 22.79% of the total effect. The mediating effect includes two indirect effects: (1) the first is the mediating role of PMPT (0.041, 19.07% of the total effect); (2) the second is the chain mediating effect of PBJW–PMPT (0.008, 3.72% of the total effect).

**Table 2:** Bootstrap analysis of mediating effects test.

Influence path	Standardized indirect effect estimation	95% CI	
		Lower limit	Upper limit
Parental emotional warmth→PMPT→IAB	0.332×0.123=0.041	0.000	0.078
Parental emotional warmth→BJW→PMPT→IAB	0.364×0.180×0.123=0.008	0.002	0.022

## Discussion

### Direct Effect of PEM on College Students’ IAB

The results of this study found that PEW had a significant positive predictive effect on college students’ IAB, which is consistent with previous studies (Padilla-Walker et al., 2016). Children accepted by parents generally demonstrate the behaviors needed by society, such as emotional stability and compassion. When parents provide additional respect, understanding, and emotional warmth to their children, the development of children’s prosocial behavior will increase, and there will be an increase in altruistic behavior when dealing with problems encountered on the Internet.

### Mediating Effects of PBJW and PMPT

The results of this study showed that PEW had an indirect effect on college students’ IAB through PMPT, validating our research hypothesis. According to social learning theory, during the socialization process, individuals acquire some behavioral norms mainly through the identification of examples and focus considerably on imitating examples with similar characteristics (Liu et al., 2003). Thus, individuals who grow up under PEW imitate their parents’ emotional warmth and upbringing behavior, thereby forming PMPT. Individuals with PMPT are often enthusiastic, friendly, and good in interpersonal communication. Studies have shown that individuals who like to socialize show increased altruistic behavior (Liu and Yang, 2004). Therefore, PMPT plays a significant mediating role in the impact of PEW on college students’ IAB.

However, there was no mediating effect of PBJW on the relationship between PEW and IAB, which is inconsistent with the results of previous studies (Jiang et al., 2017; Quan, 2021). A possible reason is that in some respects, IAB is different from altruistic behavior in real life. In the real world, mutual helping is regarded as a social norm that expresses the principles of fairness. However, cyberspace is accompanied by anonymity; thus, IAB is almost unidirectional. Hence, individuals with PMPT prefer to engage in IAB.

The results of this study showed that PEW had an indirect effect on college students’ IAB through PBJW and PMPT. Parents’ positive educational behaviors and effective interactions with children (i.e., active parenting styles) may promote the development of children’s PBJW. The warm support and reasonable feedback of positive parenting styles provide a good family atmosphere for children and adolescents. If parents treat children as independent individuals equally and fairly, the children’s PBJW will improve. Individuals with a strong PBJW are likely to form positive and unselfish PMPT. Therefore, PBJW and PMPT play a significant intermediary role in the impact of PEW on college students’ IAB.

### Limitations

First, this cross-sectional study only examined correlations of values and could not provide causal interpretations. It is thus important for longitudinal studies to replicate our findings in the future. Second, the questionnaires in this study were self-reported. Although, the common method bias test was used, future studies may attempt to use the other-report method to test the effects of mediation factors on the mechanisms of IAB. Third, the sample of this study was from two colleges; thus, samples in different regions and age groups can be used for future research.

## Conclusion

In summary, the present study emphasized the role of parenting style, PBJW, and PMPT in improving engagement in IAB among Chinese college students. PBJW is an important cognitive value that influences the behavior of individuals. Parents and educators should adopt a positive parenting style and create a fair environment to improve children’s PBJW. Our findings also have implications for the cultivation of PMPT. In addition, considering the overall mediating effect of moral personality on PBJW and IAB, parents and educators should attach importance to the cultivation of moral personality to stimulate participation in IAB.

## Data Availability Statement

The datasets presented in this study can be found in online repositories. The names of the repository/repositories and accession number(s) can be found at: http://dx.doi.org/10.17632/2jbwvx2my7.1.

## Ethics Statement

The studies involving human participants were reviewed and approved by The Research Ethics Committee of the University of Science and Technology Liaoning (China). The patients/participants provided their written informed consent to participate in this study.

## Author Contributions

YZ: conceptualization, validation, investigation, resources, and writing – original draft preparation. LC: methodology, software, and formal analysis. YX: methodology, writing – review and editing. YZ and LC: data curation, writing – review and editing, and funding acquisition. All authors contributed to the article and approved the submitted version.

## Funding

This research was funded by National Social Science Foundation of China (BHA180128), Liaoning Social Science Planning Foundation (L20ASH005), General Program of Humanities and Social Sciences Research of Department of Education of Liaoning Province (2020LNQN08), and University of Science and Technology Liaoning Talent Project Grants (601011507-33).

## Conflict of Interest

The authors declare that the research was conducted in the absence of any commercial or financial relationships that could be construed as a potential conflict of interest.

## Publisher’s Note

All claims expressed in this article are solely those of the authors and do not necessarily represent those of their affiliated organizations, or those of the publisher, the editors and the reviewers. Any product that may be evaluated in this article, or claim that may be made by its manufacturer, is not guaranteed or endorsed by the publisher.

## References

[ref1] Bègue L. Muller D. (2006). Belief in a just world as moderator of hostile attributional bias. Br. J. Soc. Psychol. 45, 117–126. doi: 10.1348/014466605X37314, PMID: 16573876

[ref2] Dalbert C. (1999). The world is more just for me than generally: about the personal belief in a just world scale's validity. Soc. Justice Res. 12, 79–98. doi: 10.1023/A:1022091609047

[ref3] Dalbert C. Sallay H. (Eds.) (2004). The Justice Motive in Adolescence and Young Adulthood: Origins and Consequences Routledge.

[ref4] Fatima S. Dawood S. Munir M. (2020). Parenting styles, moral identity and prosocial behaviors in adolescents. Curr. Psychol. doi: 10.1007/s12144-020-00609-3

[ref5] Gauvain M. Huard R. D. (1999). Family interaction, parenting style, and the development of planning. J. Fam. Psychol. 13, 75–92. doi: 10.1037/0893-3200.13.1.75

[ref6] Grusec J. E. Goodnow J. J. (1994). Impact of parental discipline methods on the child's internalization of values: a reconceptualization of current points of view. Dev. Psychol. 30, 4–19. doi: 10.1037/0012-1649.30.1.4

[ref7] Hafer C. L. Sutton R. M. (2016). “Belief in a just world” in Handbook of Social Justice Theory and Research. eds. Sabbagh C. Schmitt M. (New York: Springer).

[ref8] Homans G. C. (1958). Social behavior as exchange. Am. J. Sociol. 63, 597–606. doi: 10.1086/222355

[ref9] Jiang J. (2004). Parental rearing pattern and behavior problem in adolescents. Health Psychol. J. 12, 72–74. doi: 10.3969/j.issn.1005-1252.2004.01.031

[ref10] Jiang H. Chen G. Wang T. (2017). Relationship between belief in a just world and internet altruistic behavior in a sample of Chinese undergraduates: multiple mediating roles of gratitude and self-esteem. Pers. Individ. Differ. 104, 493–498. doi: 10.1016/j.paid.2016.09.005

[ref11] Jiang J. Lu Z. R. Jiang B. Q. Xu Y. (2010). Revision of the short-form egna minnen beträffande uppfostran for Chinese. Psychol. Dev. Educ. 6, 94–99. doi: 10.3969/j.issn.1672-3791.2014.23.102

[ref12] Khaleque A. (2013). Perceived parental warmth, and children's psychological adjustment, and personality dispositions: a meta-analysis. J. Child Fam. Stud. 22, 297–306. doi: 10.1007/s10826-012-9579-z

[ref13] Khera M. L. K. Harvey A. J. Callan M. J. (2014). Beliefs in a just world, subjective well-being and attitudes towards refugees among refugee workers. Soc. Justice Res. 27, 432–443. doi: 10.1007/s11211-014-0220-8

[ref14] Lerner M. J. Miller D. T. (1978). Just world research and the attribution process: looking back and ahead. Psychol. Bull. 85, 1030–1051. doi: 10.1037/0033-2909.85.5.1030

[ref15] Lerner M. J. Simmons C. H. (1966). Observer’s reaction to the ‘innocent victim’: compassion or rejection? J. Pers. Soc. Psychol. 4, 203–210. doi: 10.1037/h0023562, PMID: 5969146

[ref16] Li D. (2000). A study of factors influencing the prosocial behavior of children. J. Psychol. Sci. 23, 285–289.

[ref17] Liu W. Yang L. Z. H. (2004). Study on the relations among children's social inhibition, parenting and altruism. Psychol. Dev. Educ. 20, 6–11. doi: 10.3969/j.issn.1001-4918.2004.01.002

[ref18] Liu Z. H. J. Zhang Y. Tan Q. B. (2003). Senior students’ self-concept, parents’ rearing style and their prosocial behavior. J. Xiangtan Norm. Univ. 25, 112–115.

[ref19] Nudelman G. (2013). The belief in a just world and personality: a meta-analysis. Soc. Justice Res. 26, 105–119. doi: 10.1007/s11211-013-0178-y

[ref20] Padilla-Walker L. M. Nielson M. G. Day R. D. (2016). The role of parental warmth and hostility on adolescents’ prosocial behavior toward multiple targets. J. Fam. Psychol. 30, 331–340. doi: 10.1037/fam0000157, PMID: 26414417

[ref21] Perris C. Jacobsson L. LinndstrőM H. Knorring L. Perris H. (1980). Development of a new inventory for assessing memories of parental rearing behaviour. Acta Psychiatr. Scand. 61, 265–274. doi: 10.1111/j.1600-0447.1980.tb00581.x, PMID: 7446184

[ref22] Quan S. (2021). Socioeconomic status and prosocial behaviors among Chinese emerging adults: sequential mediators of parental warmth and personal belief in a just world. Child. Youth Serv. Rev. 120:105680. doi: 10.1016/j.conbuildmat.2005.08.001

[ref23] Ran B. Che H. Hui Y. (2007). Item parceling strategies in structural equation modeling. Constr. Build. Mater. 21, 7–11. doi: 10.1016/j.childyouth.2020.105680

[ref24] Sproull L. Conley C. A. Moon J. Y. (2005). “Pro-social behavior online” in The Social Net: Understanding our Online Behavior. ed. Amichai-Hamburger Y. (Oxford, UK: Oxford University Press), 139–161.

[ref25] Sutton R. M. Winnard E. J. (2007). Looking ahead through lenses of justice: the relevance of just-world beliefs to intentions and confidence in the future. Br. J. Soc. Psychol. 46, 649–666. doi: 10.1348/014466606X166220, PMID: 17877857

[ref26] Takebe M. Murata K. (2017). Perceived autonomous help and recipients' well-being: is autonomous help good for everyone? Curr. Res. Soc. Psychol. 25, 56–65.

[ref27] Wang Y. Q. (2009). Research on the Structure, Characteristics and Intervention of Moral Personality of College Students. Doctoral dissertation, Nanjing Normal University.

[ref001] Wang Y. Q. Guo B. Y. (2013). A preliminary study on the characteristics of undergraduates’ moral personality. J. Psychol. Sci. 6, 1436–1440.

[ref28] Wang H. Wang Y. Nie J. Lei L. (2021). Family socioeconomic status and internet altruistic behavior among Chinese adolescents: the mediating effect of personal belief in a just world and emotional intelligence. Child. Youth Serv. Rev. 121:105841. doi: 10.1016/j.childyouth.2020.105841

[ref29] Wolfradt U. Engelmann S. (2003). Perceived parenting styles, depersonalisation, anxiety and coping behaviour in adolescents. Pers. Individ. Differ. 34, 521–532. doi: 10.1016/S0191-8869(02)00092-2

[ref30] Wu M. S. Yan X. Zhou C. H. Chen Y. Li J. Zhu Z.. (2011). General belief in a just world and resilience: evidence from a collectivistic culture. Eur. J. Personal. 25, 431–442. doi: 10.1002/per.807

[ref31] Zheng X. L. Gu H. G. (2012). Personality traits and internet altruistic behavior: the mediating effect of self-esteem. Chin. J. Spec. Educ. 2, 69–75.

[ref33] Zheng X. L. Zhu C. L. Gu H. G. (2011). Development of internet altruistic behavior scale for college students. Chinese J. Clin. Psychol. 19, 606–608.

